# Research on Positioning Method in Underground Complex Environments Based on Fusion of Binocular Vision and IMU

**DOI:** 10.3390/s22155711

**Published:** 2022-07-30

**Authors:** Jie Cheng, Yinglian Jin, Zhen Zhai, Xiaolong Liu, Kun Zhou

**Affiliations:** 1 School of Mechanical and Electrical Engineering, China Jiliang University, Hangzhou 310018, China; cj1426163582@163.com (J.C.); zhaizhen2022@163.com (Z.Z.); 2 College of Modern Science and Technology, China Jiliang University, Hangzhou 310018, China; jinyinglian@cjlu.edu.cn; 3 Department of Mechanical Engineering, Johns Hopkins University, Baltimore, MD 21211, USA; xiaolong@jhu.edu

**Keywords:** SLAM (simultaneous localization and mapping), sensor fusion of visual and IMU, nonlinear optimization, sliding window

## Abstract

Aiming at the failure of traditional visual slam localization caused by dynamic target interference and weak texture in underground complexes, an effective robot localization scheme was designed in this paper. Firstly, the Harris algorithm with stronger corner detection ability was used, which further improved the ORB (oriented FAST and rotated BRIEF) algorithm of traditional visual slam. Secondly, the non-uniform rational B-splines algorithm was used to transform the discrete data of inertial measurement unit (IMU) into second-order steerable continuous data, and the visual sensor data were fused with IMU data. Finally, the experimental results under the KITTI dataset, EUROC dataset, and a simulated real scene proved that the method used in this paper has the characteristics of stronger robustness, better localization accuracy, small size of hardware equipment, and low power consumption.

## 1. Introduction

With the continuous expansion of urban scale and the continuous development of urban underground space, urban underground complex plays a more and more important role in urban development. At present, urban underground complexes mainly include rail transit stations, underground commercial streets, underground parking lots, and underground building entrances [1]. Underground commercial streets are places with high fire incidence, and the exhaust gas concentration of an underground parking lot is high. It is very important to design mobile robots to replace manual inspection in underground complex. The synchronous location and mapping (SLAM) technology is the key to realizing the autonomous navigation and inspection of mobile robots. Slam technology aims to use the self-loaded sensors to estimate their own position and posture, as well as to dynamically build a real-time map of the surrounding environment in an incremental way. After nearly 20 years of development, slam technology has played an important role in the fields of autonomous driving, unmanned driving, virtual reality, augmented reality, unmanned aerial vehicles, and so on. At present, the sensors used in the slam system mainly include camera, lidar, and inertial measurement units (IMU), and have two directions: laser slam and visual slam.

In 2014, Zhang and Singh proposed the LOAM (LIDAR odometry and mapping) algorithm [2], which obtained better pose estimation and 3D maps by calculating the curvature of each point in the point cloud and extracting the plane and edge features in the point cloud. In 2016, Google opened the laser SLAM algorithm Cartographer [3], which proposed the idea of local submap, which greatly accelerated the speed of loop-closure. In 2018, Shan and others proposed the Lego-LOAM [4], which proposed the idea of clustering based on LOAM. In inter frame matching, only the points of the same cluster other than the ground cluster are matched, which increases the processing efficiency and ensures high accuracy. In the same year, Kim and others proposed SC-Lego-LOAM and added the Scan Context algorithm [5] on the basis of Lego-LOAM, which made up for the defect of poor loop-closure effect of Lego-LOAM. In 2020, Shan and others proposed the LIO-SAM algorithm [6]. LIO-SAM is a tightly coupled lidar inertial odometer framework based on factor graph, which is suitable for multi-sensor fusion and global optimization. It proposes an efficient scanning matching method based on the local sliding window, which improves the real-time performance. The above methods [2,3,4,5,6] belong to laser-based SLAM. LSD-SLAM (large-scale direct monocular SLAM) [7] proposed by Engel and others. It is a SLAM system based on direct method. The system is suitable for large-scale scenes and building large-scale and globally consistent environment maps. The visual odometer based on DSO (direct spark odometry) [8] sparse direct method is superior to LSD-SLAM in accuracy, stability, and speed. The semi-direct visual odometer SVO (semi-direct visual odomtry) was proposed by Forster et al. [9]. It combines the advantages of the method based on feature points and the direct tracking optical flow method, generating a lightweight environment map. The ORB-SLAM framework is a popular visual framework of the feature point method at present [10]. The front end is based on ORB features, which not only reduce the amount of calculation but also have higher accuracy than the direct method. On the basis of this framework, researchers have proposed several improved versions. ORB-SLAM2 is the first open-source SLAM algorithm supporting monocular, binocular, and RGB-D cameras; the BA method used at the back end of the algorithm can accurately estimate the scale of the trajectory [11]. Carlos and others proposed ORB-SLAM3 in 2021 [12]. The multi-map system of the algorithm enabled the algorithm to maintain operation when visual information was lost for a long period of time. The above methods belong to visual-SLAM. However, there are a large number of mobile people in the underground complex, which seriously interferes with the information collected by laser sensors and visual sensors. The texture of the building facing materials of the underground complex is simple, and the environmental features are sparse, so it is difficult to extract the features. As a result, the traditional laser slam and visual slam are difficult to be directly applied to the scene of an underground complex.

Aiming at the dynamic target interference and weak texture characteristics of underground complex, a robot positioning method based on binocular vision and inertial navigation (IMU) fusion is designed in this paper.

The main contributions of this paper are provided as follows:

(1) The combination of the Harris algorithm and the optical flow pyramid tracking method enhances the robustness of the visual positioning system and then solves the problem of the front end being difficult in terms of extracting feature points in weak texture and it being easy to lose tracking.
(2) The interpolation algorithm of non-uniform rational B-spline is used to transform the discrete data of IMU into a continuous track that can be second-order differentiated, to align the visual and inertial information, and to improve the anti-dynamic interference ability by integrating the visual and inertial data.

The remaining parts of this paper are organized as follows: Section 2 mainly introduces the hardware framework. Section 3 presents the core algorithm design of binocular vision and interval navigation fusion. Some practical experiments are presented in Section 4 to demonstrate the effectiveness of the obtained results. The last section concludes this paper.

## 2. Hardware Framework

**Figure 1:**
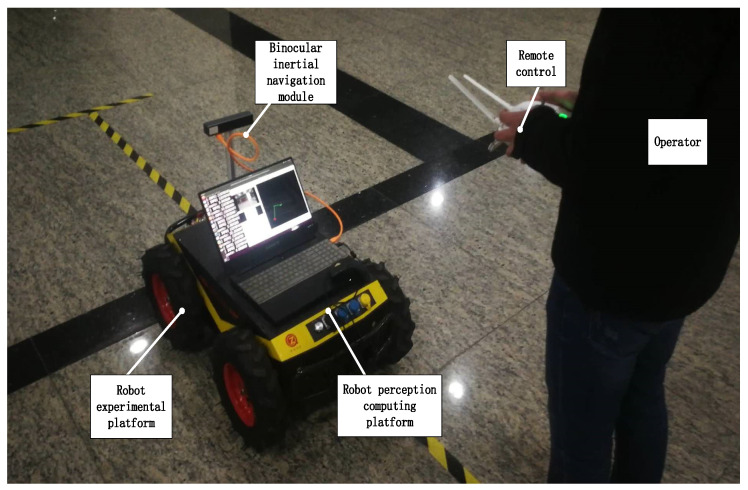
Hardware framework of robot.

As shown in Figure 1, the hardware framework used in this paper is mainly composed of the binocular inertial navigation module, the inspection robot (chassis), the remote control, and the robot perception computing platform.

The camera uses the small binocular inertial navigation integrated module and uses a 120 degree wide angle lens to obtain environmental information as much as possible. The IMU is a six-axis BMI088 sensor manufactured by BOSCH with a synchronization accuracy of 0.05 ms.

The computing platform is mainly responsible for processing sensor data, using Intel(R) Core(TM) I7-7700HQ CPU, 4 cores, 8 threads, 16 GB LPDDR4 memory; the main frequency is 2.80 ghz. The operating system is Ubuntu18.04 LTS, in which ROS (robot operating system) is installed to deal with the communication of various sensor data and the operation of specific algorithms.

## 3. Core Algorithm Design of Binocular Vision and Interval Navigation Fusion

In this paper, the positioning method of visual fusion inertial navigation designed is mainly composed of the following three parts:

(1) Through the study of traditional visual odometers, it is found that the front end based on ORB feature points is not suitable for a weakly textured environment. The improved Harris algorithm with a stronger ability to extract corner points ensures that the front end can extract more feature points from weak texture.
(2) Due to the high sampling frequency of the inertial navigation system, it is difficult to align and fuse high-frequency data with low-frequency data. To solve these problems, firstly, we studied and analyzed various factors of inertial navigation error, simplified the inertial navigation model, and reduced the difficulty of calculation. Secondly, non-uniform rational B-spline algorithm was used to transform high-frequency discrete IMU data into a second-derivable continuous trajectory, which is convenient for fusion with visual information.
(3) As for integration of vision and inertial navigation, first, since every update of back-end optimization needs to recalculate the inertial derivative data in the world coordinate system, the original inertial derivative data can be preprocessed to obtain the pre-integral, which can reduce the number of repeated calculation and deduce the Jacobian matrix and covariance. Secondly, visual and inertial navigation information were integrated in a tightly coupled way to construct optimization objective function and state variables. Thirdly, a more reasonable Levenberg–Marquardt method was introduced to solve the non-singular ill-condition problems in the fusion optimization problem. Fourthly, sliding windows and marginalization were adopted to delete the old state quantity and release the computing power in view of the growing computing scale of the back end.

**Figure 2:**
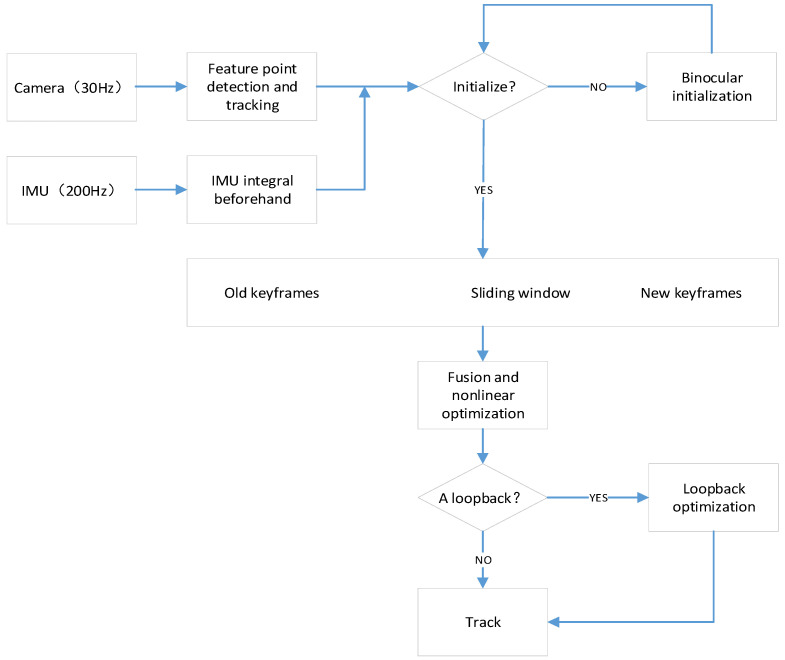
The algorithmic framework used in this paper.

The above steps can be represented by the following flow chart (Figure 2):

### 3.1. Binocular Vision Algorithm

In this paper, the binocular vision algorithm mainly consisted of front end, back end, and loop-closure.

In the front end, the following method was used to obtain the camera pose transformation between two adjacent frames:

(1) Extract Harris feature points of the first frame [13].
(2) LK multilayer optical flow was used to calculate the corresponding feature points in the second frame [14].
(3) DLT (direct linear transformation) was used to calculate the pose transformation of the camera through matched pairs of feature points [15].

In the back end, BA (bundle adjustment method) was adopted to modify the existing camera pose and roadmap [16].

In the aspect of loop-closure, the bag-of-words (BoW) model was adopted, and the steps are as follows:

(1) A number of pictures around the field scene were collected in advance, FAST feature points of each image were extracted, BRIEF descriptors of feature points were calculated to construct words of the image [17], DBoW3 was used as a dictionary to store words [18,19], and the TF-IDF algorithm was used to add different weights to different words [20].
(2) At the same time, for each image obtained, the characteristics of the image were described as words in BoW, the frequency of words was counted, and an image was converted into a group of words.

The similarity of words in each image is calculated to determine whether loop-closure is triggered.

### 3.2. Inertial Navigation Algorithm

The inertial navigation system has a stronger anti-interference ability compared with the visual positioning system, but there are many factors affecting the error that will lead to the drift of the inertial measurement value and the accumulated error being too large. This paper introduces the basic content of inertial navigation and lays the foundation for the subsequent multi-sensor fusion.

#### 3.2.1. Inertial Error Model

The errors of the accelerometer and gyroscope in inertial navigation (IMU) can be divided into deterministic errors and random errors. Deterministic errors can be obtained through prior calibration, which mainly includes bias errors, scale errors, and axis misalignments [21]. For random error, it is assumed that the noise obeys Gaussian distribution, including Gaussian white noise and bias random walk. The formula of the total error model can be expressed as

(1) $${\tilde{\omega }}^{b}=S_{g}\omega^{b}+b^{g}+n^{g}$$

(2) $${\tilde{a}}^{b}=S_{a}q_{bw}\left(a^{w}-g^{w}\right)+b^{a}+n^{a}$$

In Formula (1), ${\tilde{\omega }}^{b}$ and $\omega^{b}$ represent the measured value and the real value of angular velocity in the body coordinate system of IMU, respectively; $b^{g}$ and $n^{g}$ denote the bias random walk and Gaussian white noise of the gyroscope, respectively; and $S_{g}$ is the scale factor of the gyroscope. In Formula (2), ${\tilde{a}}^{b}$ is the measured value of acceleration in the body coordinate system of IMU; $a^{w}$ and $g^{w}$ are the world coordinates of real value and gravity acceleration, respectively; $b^{a}$ and $n^{a}$ denote the bias random walk and Gaussian white noise of the accelerometer, respectively; $S_{a}$ is the scale factor of the accelerometer; and $q_{bw}$ denotes the rotation transformation from body to world coordinates.

#### 3.2.2. Inertial Derivatives Calculated

There are usually two methods to solve IMU data, namely, the Euler method and the median method.

The Euler method assumes that the derivative of the integrand function is constant in a short period of time. When calculating the pose at *k*+1, the linear velocity and angular velocity used are those measured at *k*, and the formula can be designed as

(3) $$P_{wb_{k+1}}=P_{wb_{k}}+v_{k}^{w}\Delta t+\frac{1}{2}a\Delta t^{2}$$

(4) $$v_{k+1}^{w}=v_{k}^{w}+a\Delta t\;$$

(5) $$q_{wb_{k+1}}=q_{wb_{k}}⊗\left[\begin{array}{c} 1\\ \frac{1}{2}\omega \delta t \end{array}\right]\;$$

(6) $$a=q_{wb_{k}}\left(a^{b_{k}}-b_{k}^{a}\right)-g^{w}$$

(7) $$\omega =\omega^{b_{k}}-b_{k}^{g}\;$$

In the above formula, $P_{wb_{k}}$ and $P_{wb_{k+1}}$ are the position coordinates of the robot in the world coordinate system at time *k* and time *k*+1, respectively; $v_{k}^{w}$ and $v_{k+1}^{w}$ are the robot speed in the world coordinate system at time *k* and time *k*+1, respectively; a is the acceleration; Δt is the time interval between *k* and *k*+1; $q_{wb_{k}}$ and $q_{wb_{k+1}}$ denote robot rotation information in the world coordinate system at time *k* and time *k*+1, respectively; ⊗ stands for quaternion multiplication; $a^{b_{k}}$ is the acceleration in the body coordinate system of IMU at time *k*; $b_{k}^{a}$ is the bias noise; $g^{w}$ denotes acceleration of gravity; $\omega^{b_{k}}$ is the angular velocity in the body coordinate system of IMU at time *k*; and $b_{k}^{g}$ denotes the bias noise.

Although the median method also assumes that the derivative of the integrand is constant in a short period of time, the linear velocity and angular velocity used in the calculation are the average values of the linear velocity and angular velocity measured at the moment *k* and *k*+1, and the formula can be expressed as

(8) $$P_{wb_{k+1}}=P_{wb_{k}}+v_{k}^{w}\Delta t+\frac{1}{2}a\Delta t^{2}$$

(9) $$v_{k+1}^{w}=v_{k}^{w}+a\Delta t\;$$

(10) $$q_{wb_{k+1}}=q_{wb_{k}}⊗\left[\begin{array}{c} 1\\ \frac{1}{2}\omega \delta t \end{array}\right]\;$$

(11) $$a=\frac{1}{2}\left[q_{wb_{k}}\left(a^{b_{k}}-b_{k}^{a}\right)-g^{w}+q_{wb_{k+1}}\left(a^{b_{k+1}}-b_{k}^{a}\right)-g^{w}\right]\;$$

(12) $$\omega =\frac{1}{2}\left[\left(\omega^{b_{k}}-b_{k}^{g}\right)+\left(\omega^{b_{k+1}}-b_{k}^{g}\right)\right]\;$$

The new variables in the above formula are as follows: $a^{b_{k+1}}$ is the acceleration in the body coordinate system of IMU at *k*+1, and $\omega^{b_{k+1}}$ is the angular velocity in the body coordinate system of IMU at *k*+1.

Inertial navigation has a very high sampling frequency (usually between 100 and 200 Hz) and the raw data obtained from inertial navigation is huge. The computing platform mounted on the inspection robot has limited computing power, but it is expected that the calculation error is small. Therefore, a simulation experiment was designed to compare the performance of the two algorithms.

Suppose a particle is moving elliptically in the xy plane and sinusoidal in the z axis. The other particle performs the same motion as the first point, but adds the IMU kinematic model and outputs the values measured by the IMU in the simulation. According to the motion model of IMU in continuous time, the Euler method and median method are adopted. By comparing the error mean and time consumption of the Euler method and median method, an appropriate integration algorithm is selected.

Let the motion parameter equation of the particle be translated as

(13) $$\left(15\cos\left(\pi t/10+5\right),\;\;20\sin\left(\pi t/10\right),\;\;\sin\left(\pi t\right)+5\right)$$

The rotation formula can be expressed as

(14) $$\left(0.1\cos\left(t\right),\;\;0.2\sin\left(t\right),\;\;\pi t\right)$$

where t is the time.

**Figure 3:**
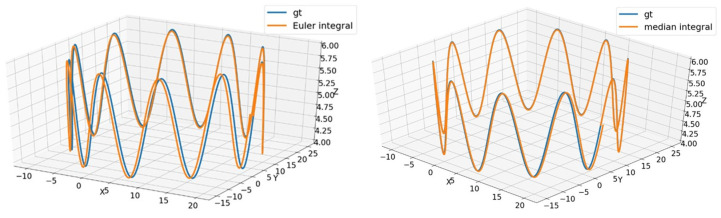
The comparison of Euler’s method (**left**) and median method (**right**).

As shown in Figure 3, the blue curve is the true value of particle trajectory, while the yellow curve is the trajectory of a particle calculated by the Euler method or median method under the measurement of the IMU model. The mean error of the Euler method is 0.03099, and the mean error of median method is 0.02130. The latter is 31.27% higher than the former. The average calculation time of the two algorithms on 10 sets of data are 0.05571 s and 0.07061 s, respectively. The speed of the former is 21.10% faster than the latter. In the fusion algorithm, even if the initial pose is not accurate, the subsequent pose will continue to be optimized. However, time wasted cannot be recovered, which is an irreversible indicator. In view of the large number of computing modules in the system, it is necessary to allocate computing resources reasonably to ensure the real-time performance of the fusion system. Therefore, for the whole visual and inertial navigation integration system, this paper finally chose the Euler integral.

#### 3.2.3. Nonuniform Rational B Spline

The discrete position, velocity, and attitude information can be obtained by IMU data solution, and the position and attitude information obtained by visual odometer is also discrete. The problem of timestamp unsynchronization exists between two kinds of discrete data [22]. If a smooth, multi-order differentiable curve is generated according to the discrete visual odometer data, the angular velocity can be obtained by taking the first derivative of the curve, which can be compared with the angular velocity obtained by IMU solution, so as to realize the alignment between the visual odometer and IMU data.

The Bessel curve is usually used to series known discrete points to generate a continuous trajectory, but when there are too many points, adjusting any one point will affect the whole Bessel curve, and each point will have weaker control over the curve. On this basis, this paper improved and adopted the NURBS (non-uniform rational B-splines) method [23] to solve the above problems through multiple recursion of piecewise functions. Non-uniform means that there is a difference between each two node vectors, and rational means that different weights can be applied to each point; the formula can be expressed as

(15) $$P\left(t\right)=\frac{\sum_{i=0}^{n}\omega_{i}P_{i}N_{i,k}\left(t\right)}{\sum_{i=0}^{n}\omega_{i}N_{i,k}\left(t\right)}$$

where t denotes the node, $T=\left[t_{0},t_{1},\ldots ,t_{n+k}\right]$ means the set range, $\omega_{i}$ means the weight factor, k is the degree of this curve, $N_{i,k}\left(t\right)$ denotes the i-th and k-th B-spline basis function, and $p_{i}$ means the i-th control point in the Bezier curve; the recursive formula (Cox-de Boor) is defined as

(16) $$N_{i,0}\left(t\right)=\left\{\begin{array}{l} 1,t_{i}\leq t<t_{i+1}\\ 0,t\geq t_{i+1}\;or\;t<t_{i+1} \end{array}\right.$$

(17) $$N_{i,k}\left(t\right)=\frac{t-t_{i}}{t_{i+k}-t_{i}}N_{i,k-1}\left(t\right)+\frac{t_{i+k+1}-t}{t_{i+k+1}-t_{i+1}}N_{i+1,k-1}\left(t\right),k\geq 1\;$$

Through the recursive logic of B-spline basis function, it can be found that each k-degree basis function only has values in n node intervals, which means that this control point achieves local control that the Bessel curve cannot achieve.

### 3.3. A Tightly Coupled Visual and Inertial Fusion Algorithm

The first two sections mainly introduce the method of pure visual positioning and pure inertial positioning and make some improvements for specific scenes. The main content of this section is to solve the problem of fusion of two types of non-homologous sensor data.

#### 3.3.1. IMU Data Preprocessing

IMU has a high sampling frequency and a large amount of data. In optimization problems, it is impossible to put so much data into state variables. Therefore, the usual practice is as follows: Given the translation, velocity, and attitude of the *k*-th s, and given all the data between the *k*-th sand the *k*+1 s as well as the known dynamics equation, integrating from the *k*-th s to the *k*+1 s, we can finally obtain the translation, velocity, and attitude of the next moment. However, in the subsequent optimization algorithm, if the iterative solution calculation updates the state quantity in this way, every time the starting point of the iteration changes, or if any state quantity is adjusted midway, it must be re-integrated in order to obtain the following trajectory. Limited computing resources cannot support such calculations. To solve this problem, the IMU pre-integration algorithm is used [24,25,26], and the formula can be defined as

(18) $$R_{w}^{b_{k}}p_{b_{k+1}}^{w}=R_{w}^{b_{k}}\left(p_{b_{w}}^{w}+v_{b_{k}}^{w}\Delta t_{k}-\frac{1}{2}g^{w}\Delta t_{k}^{2}\right)+\alpha_{b_{k+1}}^{b_{k}}$$

(19) $$R_{w}^{b_{k}}v_{b_{k+1}}^{w}=R_{w}^{b_{k}}\left(v_{b_{k}}^{w}-g^{w}\Delta t_{k}\right)+\beta_{b_{k+1}}^{b_{k}}\;$$

(20) $$q_{w}^{b_{k}}⊗q_{b_{k+1}}^{w}=\gamma_{b_{k+1}}^{b_{k}}\;$$

where $R_{w}^{b_{k}}$ is the rotation transformation from IMU coordinate system to the world coordinate system at time *k*; $q_{w}^{b_{k}}$ is the quaternion form; $P_{b_{k}}^{w}$ and $P_{b_{k+1}}^{w}$ represent the position coordinates of IMU at k moment and *k*+1 moment in the world coordinate system, respectively; $v_{b_{k}}^{w}$ and $v_{b_{k+1}}^{w}$ represent the velocity of IMU at time *k* and time *k*+1 in the world coordinate system, respectively; $g^{w}$ is the acceleration of gravity in the world coordinate system; and $\Delta t_{k}$ is the interval between *k* moment and *k*+1 moment; the definition of $\alpha_{b_{k+1}}^{b_{k}}$, $\beta_{b_{k+1}}^{b_{k}}$, and $\gamma_{b_{k+1}}^{b_{k}}$ are defined as follows

(21) $$\alpha_{b_{k+1}}^{b_{k}}=\iint_{t\in \left[k,k+1\right]}\left[R_{t}^{b_{k}}\left({\tilde{a}}_{t}-b_{a_{t}}\right)\right]dt^{2}$$

(22) $$\beta_{b_{k+1}}^{b_{k}}=\int_{t\in \left[k,k+1\right]}\left[R_{t}^{b_{k}}\left({\tilde{a}}_{t}-b_{a_{t}}\right)\right]dt\;$$

(23) $$\gamma_{b_{k+1}}^{b_{k}}=\int_{t\in \left[k,k+1\right]}\frac{1}{2}\Omega \left({\tilde{\omega }}_{t}-b_{w_{t}}\right)\gamma_{t}^{b_{k}}dt\;$$

(24) $$\Omega \left(\omega \right)=\left[\begin{array}{cc} -{\left[\omega \right]}_{\times } & \omega \\ -\omega^{T} & 0 \end{array}\right]\;$$

where ${\tilde{a}}_{t}$ and ${\tilde{\omega }}_{t}$ are acceleration and angular velocity given by IMU at time *T*, respectively; $b_{a_{t}}$ and $b_{w_{t}}$ are bias noises; and $\alpha_{b_{k+1}}^{b_{k}}$, $\beta_{b_{k+1}}^{b_{k}}$, and $\gamma_{b_{k+1}}^{b_{k}}$ are the pre-product components of translation, velocity, and attitude from frame $b_{k}$ to frame $b_{k+1}$ respectively.

IMU pre-integration can effectively reduce the amount of calculations. However, the noise variance of the original IMU data as the measured value can be obtained through calibration, but the uncertainty of the pre-integration is unknown now, so the covariance of the pre-integration needs to be derived.

In discrete form, the linear transfer of the incremental error at adjacent moments satisfies the equation and can be expressed as

(25) $$\delta {\dot{z}}_{t+\delta t}^{b_{k}}=F_{t}\delta z_{t}^{b_{k}}+G_{t}n_{t}$$

The parameters are as follows

(26) $$z_{t}^{b_{k}}={\left[\begin{array}{ccccc} \delta \alpha_{k} & \delta \theta_{k} & \delta \beta_{k} & \delta b_{a_{k}} & \delta b_{w_{k}} \end{array}\right]}^{T}$$

(27) $$n_{t}={\left[\begin{array}{cccccc} n_{a_{k}} & n_{w_{k}} & n_{a_{k+1}} & n_{w_{k+1}} & n_{b_{a}} & n_{b_{w}} \end{array}\right]}^{T}\;$$

(28) $$F_{t}=\left[\begin{array}{ccccc} I & f_{01} & I\delta t & f_{03} & f_{04}\\ 0 & f_{11} & 0 & 0 & -I\delta t\\ 0 & f_{21} & I & f_{23} & f_{24}\\ 0 & 0 & 0 & I & 0\\ 0 & 0 & 0 & 0 & I \end{array}\right]\;$$

(29) $$G_{t}=\left[\begin{array}{cccccc} v_{00} & v_{01} & v_{02} & v_{03} & 0 & 0\\ 0 & -\frac{I\delta t}{2} & 0 & -\frac{I\delta t}{2} & 0 & 0\\ -\frac{R_{k}\delta t}{2} & v_{21} & -\frac{R_{k+1}\delta t}{2} & v_{23} & 0 & 0\\ 0 & 0 & 0 & 0 & I\delta t & 0\\ 0 & 0 & 0 & 0 & 0 & I\delta t \end{array}\right]\;$$

where $z_{t}^{b_{k}}$ is a vector composed of five state increments, namely, pre-integrated translation, rotation, velocity, accelerometer bias noise, and gyroscope bias noise [27]. The details of parameters in matrix $F_{t}$, $G_{t}$ are shown in Table 1.

It can be seen from Formula (25) that error transmission can be divided into two types: error transmission from the current moment to the next moment and measurement noise transmission from the current moment to the next moment.

Here, $\delta z_{t+\delta t}^{b_{k}}$ can be approximated by first-order Taylor, and the formula can be expressed as

(30) $$\begin{array}{c} \delta z_{t+\delta t}^{b_{k}}=\delta z_{t}^{b_{k}}+\delta {\dot{z}}_{t}^{b_{k}}\delta t\\ \;\;\;\;\;\;\;\;\;\;\;\;\;\;\;\;\;\;\;\;\;\;\;\;\;\;\;\;\;\;\;=\left(I+F_{t}\delta t\right)\delta z_{t}^{b_{k}}+\left(G_{t}\delta t\right)n_{t}\\ \;\;\;\;\;\;\;\;\;=F\delta z_{t}^{b_{k}}+Vn_{t} \end{array}$$

According to Formula (30), IMU measurement error is transitive, and the error at the current moment has a linear relationship with the covariance at the next moment, so the latter can be calculated from the former.

The calculation formula of covariance matrix can be expressed as

(31) $$P_{t+\delta t}^{b_{k}}=\left(I+F_{t}\delta t\right)P_{t}^{b_{t}}{\left(I+F_{t}\delta t\right)}^{T}+\left(G_{t}\delta t\right)Q{\left(G_{t}\delta t\right)}^{T}$$

where the initial value of variance is $P_{0}^{b_{k}}=0$, and Q is the diagonal covariance matrix of the noise term.

In addition, according to the formula, the iterative formula of the Jacobian matrix of the error term can be obtained

(32) $$J_{t+\delta t}=\left(I+F_{t}\delta t\right)J_{t}$$

where $J_{t}$ is the Jacobian matrix, and the initial value of the Jacobian matrix is the identity matrix.

The pre-integral values of IMU, covariance matrix, and Jacobian matrix of residuals are deduced above.

#### 3.3.2. Construction of Residual Equation

In order to describe the difference between the two sensors, a nonlinear objective function needs to be constructed. The objective of optimization is to make the difference as small as possible. Optimization variables include camera information and IMU information. The initial value of iteration is the imprecise initial value measured by the sensor, and then optimization is carried out on this basis, so as to ensure that the optimized variable is the global optimal as much as possible and avoid falling into the local optimal solution.

(a) The state vector

The elements of the state vector include n+1 sensor state quantity, one external parameter, and inverse depth of m+1 space path punctuation [28]. The formula can be expressed as

(33) $$X=\left[x_{0},x_{1},\ldots ,x_{n},x_{c}^{b},\lambda_{0},\lambda_{1},\ldots ,\lambda_{m}\right]$$

(34) $$x_{k}=\left[p_{b_{k}}^{w},v_{b_{k}}^{w},q_{b_{k}}^{w},b_{a},b_{g}\right]\;$$

(35) $$x_{c}^{b}=\left[p_{c}^{b},q_{c}^{b}\right]\;$$

where X is the state vector, and $x_{k}$ denotes the state quantity of the sensor in the k-th frame in the sliding window, in which there are n key frames in total. Each state quantity has five variables, namely, the translation of the k-th frame, the velocity quantity, the rotation quantity, and the deviation between the accelerometer and the gyroscope. $x_{c}^{b}$ is the external parameter from camera to IMU. The inverse depth is the inverse of the signpost depth.

(b) Inertial constraint

Define the difference between IMU position, rotation, velocity, acceleration bias, and gyro bias changes every two frames, and the formula can be expressed as


(36) $$r_{B}\left({\tilde{z}}_{b_{k+1}}^{b_{k}},X\right)=\left[\begin{array}{c} \delta \alpha_{b_{k+1}}^{b_{k}}\\ \delta \theta_{b_{k+1}}^{b_{k}}\\ \delta \beta_{b_{k+1}}^{b_{k}}\\ \delta b_{a}\\ \delta b_{g} \end{array}\right]=\left[\begin{array}{c} R_{w}^{b_{k}}\left(p_{b_{k+1}}^{w}-p_{b_{k}}^{w}-v_{b_{k}}^{w}\Delta t_{k}+\frac{1}{2}g^{w}\Delta t_{k}^{2}\right)-\alpha_{b_{k+1}}^{b_{k}}\\ 2{\left[\gamma_{b_{k+1}}^{b_{k}}⊗q_{b_{k}}^{w}{}^{-1}⊗q_{b_{k+1}}^{w}\right]}_{xyz}\\ R_{w}^{b_{k}}\left(v_{b_{k+1}}^{w}-v_{b_{k}}^{w}+g^{w}\Delta t_{k}\right)-\beta_{b_{k+1}}^{b_{k}}\\ b_{a_{b_{k+1}}}-b_{a_{b_{k}}}\\ b_{\omega_{b_{k+1}}}-b_{\omega_{b_{k}}} \end{array}\right]$$

The optimization variables in the formula are the state quantity of frame k and the state quantity of frame k+1, and there are 10 variables in total.

(c) Visual constraints

Visual residuals occur when pixels are reprojected. In order to obtain the ideal sensor pose, visual residuals need to be minimized. The coordinate value of the landmark point l observed in the camera coordinate system of frame i projected to the pixel is converted to the camera coordinate system of frame j, and the visual residual is composed of the pixel coordinate value obtained by conversion and the directly observed pixel coordinate value, and the calculation formula can be expressed as

(37) $$r_{C}\left({\tilde{z}}_{l}^{c_{j}},X\right)=\left[\begin{array}{c} \frac{x_{c_{j}}}{z_{c_{j}}}-u_{c_{j}}\\ \frac{y_{c_{j}}}{z_{c_{j}}}-v_{c_{j}} \end{array}\right]$$

where $x_{c_{j}},y_{c_{j}},z_{c_{j}}$ are the space coordinate value obtained by conversion, and $u_{c_{j}},v_{c_{j}}$ is the pixel coordinate value obtained by direct observation. The value of the feature point in frame i projected to the camera coordinate system of frame j can be defined as

(38) $$\left[\begin{array}{c} x_{C_{j}}\\ y_{C_{j}}\\ z_{C_{j}}\\ 1 \end{array}\right]=T_{bc}^{-1}T_{wb_{j}}^{-1}T_{wb_{i}}T_{bc}\left[\begin{array}{c} \frac{1}{\lambda }u_{C_{i}}\\ \frac{1}{\lambda }v_{C_{i}}\\ \frac{1}{\lambda }\\ 1 \end{array}\right]$$

where λ is the inverse depth. The optimization variables are two moment state variables, external parameters and inverse depth.

(d) Global objective function

Building the overall objective function includes prior information, visual information, and IMU constraint information, and in integrating the information of various sensors into the nonlinear optimization to obtain the optimal state vector, the formula can be expressed as

(39) $$\underset{X}{\min}\left\{{\left\|r_{p}-J_{p}X\right\|}^{2}+\sum_{k\in B}{\left\|r_{B}\left({\tilde{z}}_{b_{k+1}}^{b_{k}},X\right)\right\|}_{P_{b_{k+1}}^{b_{k}}}^{2}+\sum_{\left(l,j\right)\in C}{\left\|r_{c}\left({\tilde{z}}_{l}^{C_{j}},X\right)\right\|}_{P_{l}^{C_{j}}}^{2}\right\}$$

where $P_{l}^{C_{j}}$ is noise covariance generated by visual observation, and the noise covariance generated by IMU pre-integration is $P_{b_{k+1}}^{b_{k}}$. Covariance is a weight that regulates the confidence of IMU and visual sensors in fusion. The more noise a sensor makes, the less reliable it is. Reduce the weight of this sensor and increase the weight of another sensor.

#### 3.3.3. Nonlinear Optimization Solution

After constructing the nonlinear objective function of vision and IMU fusion, solving this equation requires knowing the global properties of the objective function, but it cannot be solved directly. In this paper, both the Levenberg–Marquardt (L-M) method and the Gauss–Newton (G-N) method were used as the gradient descent strategy. When the problem is good, the Gauss–Newton method is used. If the problem approaches pathology, the Levenberg–Marquardt method is used [29].

In the actual fusion process of vision and IMU, if disturbed, the value of a certain residual term will be much higher than that of other normal residual terms. This wrong residual term will concentrate computing resources, resulting in the lack of computing resources for the correct residual term. Incorrect residuals can be “intelligently” filtered using the Huber kernel [30]. When the error is greater than the threshold value, it will be judged as the wrong residual term, and the function of the first form must be used to limit the error growth. If the error is less than the threshold, the original form is kept, and the formula can be expressed as

(40) $$H\left(e\right)=\left\{\begin{array}{cc} \frac{1}{2}e^{2} & if\left|e\right|\leq \delta \\ \delta (\left|e\right|-\frac{1}{2}\delta ) & otherwise \end{array}\right.$$

#### 3.3.4. Sliding Windows and Marginalization

In sum, it can be found that the optimization objective function of tightly coupled fusion data increases dynamically. With the continuous movement of the robot, the state variables in the objective function increase continuously, which brings difficulties to the optimization problem. In order to control the growth of optimization scale, this paper adopted the sliding window method. The sliding window will constantly discard the oldest state variables and add new ones, and the sum of state variables in the window will remain constant. Use of multiple state windows can make state estimation more accurate, and at the same time, a robust optimization cost function can be established to reduce the influence of external points on state estimation [31]. However, if we delete the old state directly while maintaining the sliding window, the original visual and IMU constraints will be broken. In this paper, the marginal method was used to transform the constraint information into the prior distribution of variables to be optimized.

Suppose that all states of a sliding window can be defined as follows

(41) $$\Delta x=\left[\begin{array}{c} \Delta x_{m}\\ \Delta x_{n} \end{array}\right]$$

where $\Delta x_{m}$ and $\Delta x_{n}$ represent m states to be removed and n states to be retained, respectively.

Optimization problems can be expressed as

(42) $$\begin{array}{l} H\Delta x=g\\ \left[\begin{array}{cc} A & B\\ B^{T} & D \end{array}\right]\left[\begin{array}{c} \Delta x_{m}\\ \Delta x_{n} \end{array}\right]=\left[\begin{array}{c} g_{m}\\ g_{n} \end{array}\right] \end{array}$$

where ABD is the three matrix blocks of the H matrix, and $g_{m},g_{n}$ are the two vectors of the g vector.

There are a total of m+n states in the sliding window, and these m+n variables are solved as a whole. If the inverse operation of H matrix is solved directly, it will consume a large amount of computing resources. Moreover, the $\Delta x_{m}$ needs to be deleted, so there is no need to perform a second calculation. Therefore, this paper chose to eliminate the $B^{T}$ matrix block in the lower left corner, and the formula can be expressed as

(43) $$\left[\begin{array}{cc} A & B\\ 0 & D-B^{T}A^{-1}B \end{array}\right]\left[\begin{array}{c} \Delta x_{m}\\ \Delta x_{n} \end{array}\right]=\left[\begin{array}{c} g_{m}\\ g_{n}-B^{T}A^{-1}b_{m} \end{array}\right]$$

Thus, the second row of the equation becomes independent of $\Delta x_{m}$. The state variables to be retained in the sliding window can be obtained by directly solving the second line, and the formula can be expressed as

(44) $$\left(D-B^{T}A^{-1}B\right)\Delta x_{n}=g_{n}-B^{T}A^{-1}b_{m}$$

Equation (41) requires only the inverse operation of the matrix block A. In addition, the number of deleted m variables is far less than the number of retained n variables, which greatly improves the computational efficiency.

In conclusion, there are three main purposes of marginalization: (1) the amount of computation is controlled by controlling the size of the sliding window; (2) ensuring that the frames in the sliding window have sufficient parallax; (3) the amount of operation is reduced by making the matrix dimension involved in the operation smaller.

**Figure 4:**
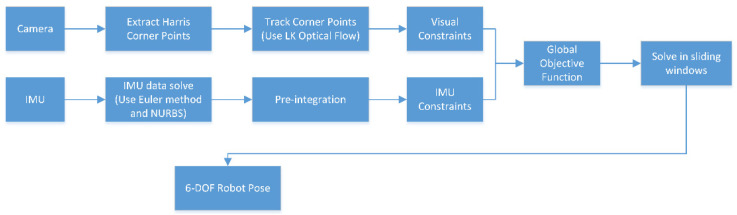
The entire process of tightly coupled visual and IMU fusion.

In addition, the entire process of tightly coupled visual and IMU fusion can be described as in Figure 4.

## 4. Experimental Results and Analysis

This chapter mainly carries on the experiment verification to the algorithm designed above. In this paper, the visual inertial navigation and positioning system were verified by dataset, quantitative positioning accuracy, and qualitative robustness experiments. Finally, the conclusion was drawn by analyzing the experimental results.

### 4.1. Experimental on Dataset

This section uses datasets to test the performance of the proposed fusion location algorithm. KITTI and EUROC datasets are mainly used in this section. The image sequence selected by the KITTI dataset was high-speed dynamic pedestrians and vehicles, with nearly 5000 images, and the scene with the longest distance up to 400 m, simulating the scene of dynamic interference in the underground complex. The image sequence selected by the EUROC dataset is the factory of weak texture, with nearly 200 images, and the scene with the longest distance of 10 m, simulating the environment of weak texture in the underground complex. The positioning system designed in this paper and ORB-SLAM2 of pure visual positioning method were tested on these two data sets. Absolute pose error was used to evaluate the global consistency of the entire trajectory.

**Figure 5:**
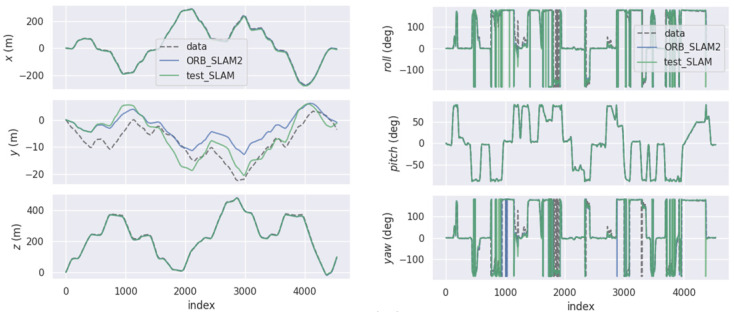
The comparison in space attitude of two algorithms in the KITTI dataset.

**Figure 6:**
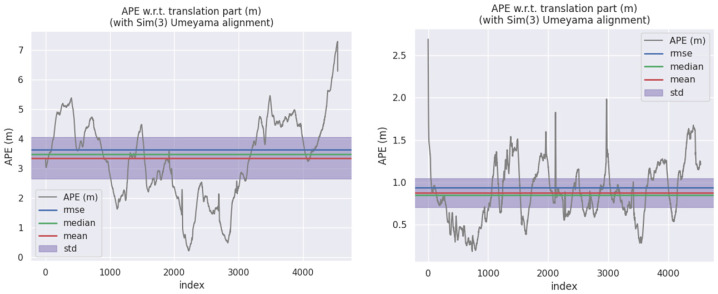
The comparison in APE of two algorithms in the KITTI dataset.

Figure 5 is the comparison of the GT (ground truth) of the KITTI dataset, the estimated value of the algorithm in this paper, and the estimated value of the pure visual method in six poses in space. The estimated data of X- and Z-axes were relatively close to the GT, while the data of the *Y*-axis had obvious deviation, the pitch angle was relatively close, and the roll angle and yaw angle had obvious deviation, but the overall trend of change was relatively consistent. As shown in Figure 6, by calculating APE, the maximum APE of ORB-SLAM2 was 7.48 m, while the maximum APE of the fusion positioning algorithm proposed in this paper was 2.76 m. It was shown that the fusion positioning system proposed in this paper can effectively reduce the errors caused by the pure visual positioning method under dynamic interference, and the improvement effect was up to 63.1%. In addition, the method adopted in this paper had lower variance and was more stable than ORB-SLAM2.

**Figure 7:**
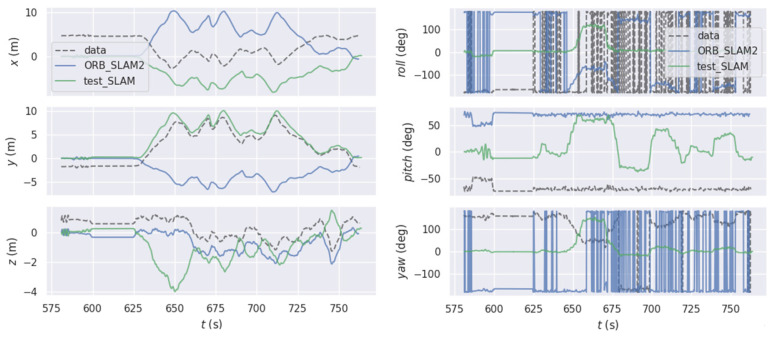
The comparison in space attitude of two algorithms in the EUROC dataset.

**Figure 8:**
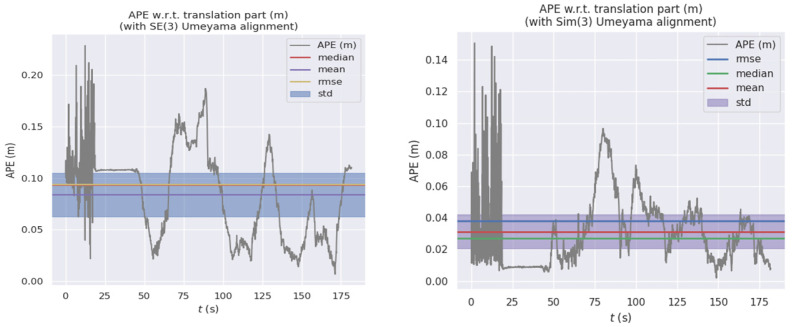
The comparison in APE of two algorithms in the EUROC dataset.

Figure 7 shows the comparison of the GT in the EUROC dataset, the estimated values of the algorithm in this paper, and the estimated values of the pure visual method in the six postures in space. Due to the serious interference of weak texture on the pure visual positioning method, the change trend of six pose was inconsistent with the GT. However, on the *X*-axis and *Y*-axis, the method used in this paper was closer to the GT than ORB-SLAM. As shown in Figure 8, by calculating APE, the maximum APE of ORB-SLAM2 was 0.238 m, while the maximum APE of the fusion positioning algorithm proposed in this paper was 0.155 m. It shows that the fusion positioning system proposed in this paper can effectively reduce the errors caused by the pure visual positioning method under the condition of weak texture, and the improvement effect was up to 34.9%.

### 4.2. Experimental in Simulated Real Scene

**Figure 9:**
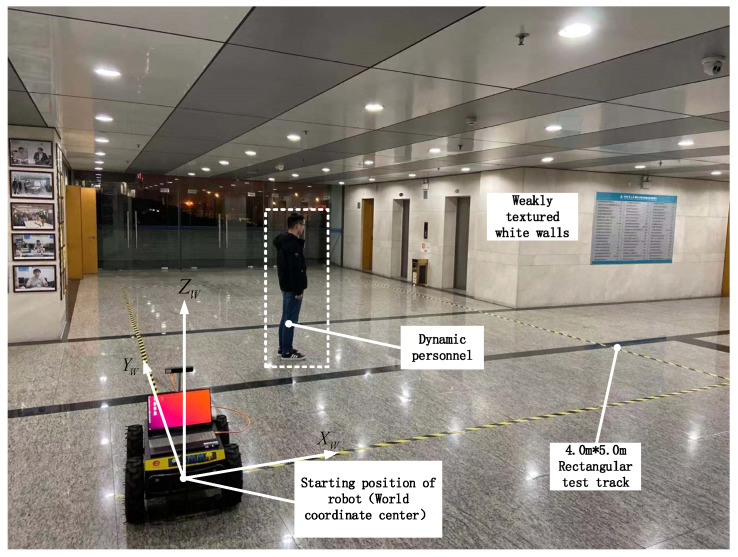
Deployment of the real environment.

In order to simulate dynamic interference and weak texture scenes of an underground complex, experiments in an underground complex involve public security and other issues. As shown in Figure 9, an indoor environment with weak texture was selected for the experiment, a number of dynamic personnel were added, and a rectangular experimental track of 4 m × 5 m was laid.

#### 4.2.1. Positioning Accuracy Test

**Figure 10:**
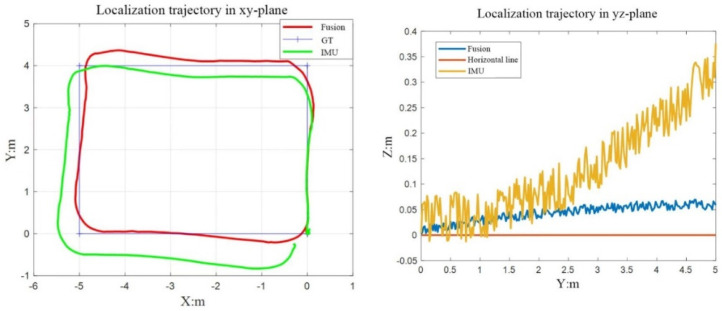
The comparison of localization accuracy between two algorithms in XOY and YOZ planes.

Due to the limitation of experimental conditions, the real pose value of the inspection robot at every moment cannot be obtained. In order to evaluate the positioning accuracy, this section conducts an evaluation from three perspectives: whether the estimated positioning trajectory was closed, the errors of the starting point and end point before and after fusion, and the trajectory deviation. A total of six groups of experiments were conducted. As shown in Figure 10, the left figure was the comparison of the positioning trajectory in the XOY plane after the pure inertial navigation and fusion. Due to the continuous accumulation of errors in the pure inertial navigation, the positioning curve was not closed, and the whole trajectory was seriously deformed. The trajectory estimated after fusion had smaller deviation and was closer to the ideal trajectory due to the addition of visual constraints. At the same time, vision can perform loop detection to make the final trajectory closed. The figure on the right shows the comparison between the pure inertial navigation and the fused positioning trajectory in the YOZ plane. The measurement results of pure inertial navigation diverged under the influence of other errors such as zero drift. The measured value of the fusion algorithm had some deviation and finally converged to a small error value.

Furthermore, Table 2 shows the statistical data of the starting point and ending point error of six groups of experiments of the two algorithms before and after fusion. The error here was measured by the distance between the two points. The average error before fusion was 0.13 m, and the average error after fusion was 0.029 m. The overall experimental effect after fusion in this paper was improved by 77.7%.

#### 4.2.2. Robustness Experiments under Dynamic Disturbances

**Figure 11:**
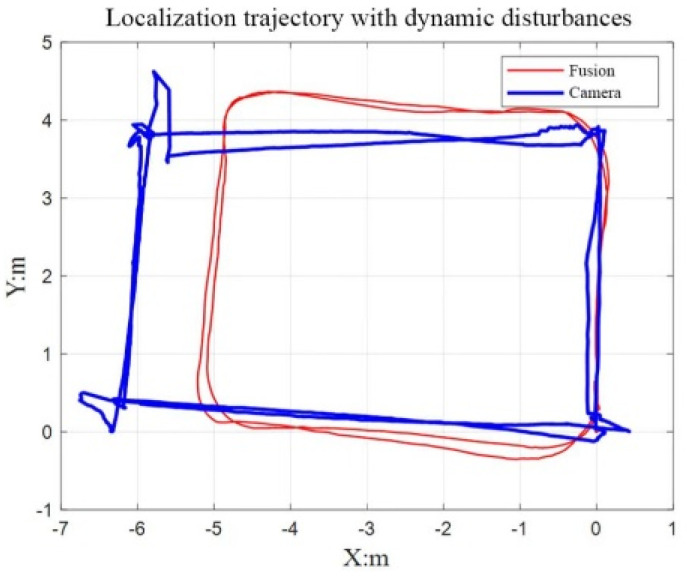
Track of two algorithms in dynamic interference.

In this experiment, the fusion localization algorithm proposed in this paper was compared with the ORB-SLAM2 algorithm of binocular pure vision under dynamic interference. We had people try three types of exercise: running, continuous walking, and intermittent walking. In the experiments of running and continuous walking, there was no significant difference between the fusion localization algorithm and the pure visual localization method, and both of them had certain robustness to interference. However, in the experiment of intermittent walking, the difference between the two algorithms was particularly obvious. As shown in Figure 11, the inspection robot was made to complete the rectangular movement of 4 × 5 m. At the same time, the experimenter continuously interfered with the binocular camera in the *X*-axis direction by intermittent walking (stopping for 20 s every 0.5 m).

**Figure 12:**
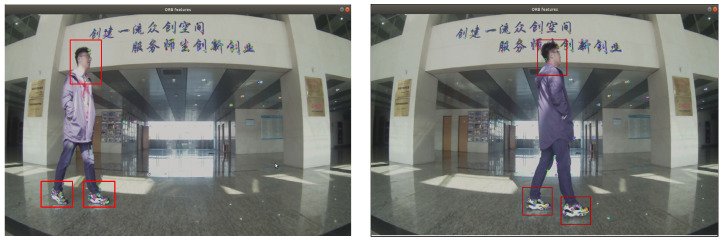
Location loss analysis.

From the experimental results, it can be found that the results of the pure visual positioning system were severely deformed, and the scale and ideal value were very different. However, the fusion localization algorithm proposed in this paper was affected by dynamic interference, and there was a certain degree of error in the localization scale, but the overall localization trajectory still maintained a smooth and flat rectangular trajectory. As shown in Figure 12, we used pure visual positioning of the ORB algorithm for image feature extraction, which can be found in the process of intermittent walking, and tester shoes and head were also full of the feature points; the ORB algorithm can extract all feature points in the environment, but not the difference between dynamic and non-rigid or static, rigid wall. When the visual sensor was still and the tester moved to another position, the visual sensor still misinterpreted the dynamic, non-rigid person as a static wall, resulting in a false displacement. As can be seen from the experimental results, the purely visual positioning method produced more displacement in the *X*-axis direction, and the own rotational motion of robot at the turning point led to more severe errors. The positioning method in this paper adopted the multi-sensor fusion method, and the inertial navigation measured the motion of the inspection robot itself, which was not affected by environmental changes. Therefore, the positioning trajectory was close to the ideal value, and the effect was better than the pure visual positioning method.

#### 4.2.3. Robustness Experiments with Missing Textures

**Figure 13:**
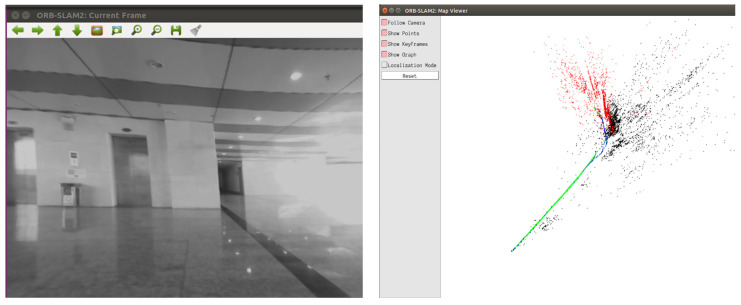
Localization results of ORB-SLAM2 in a low-texture environment.

In this experiment, the fusion localization algorithm proposed in this paper was compared with the ORB-SLAM2 algorithm of binocular pure vision in the absence of texture. The experimental environment simulated the underground complex, and the indoor environment with weak texture was selected for the experiment. As shown in Figure 13, when the inspection robot was displaced to the first corner, because the building facing smooth materials leveled off, the mirror was serious, and in some places, there was an overexposed photo; at the same time, in the face of the white wall texture feature not being obvious, on the basis of the characteristic point of the ORB-SLAM2 pure visual positioning method, it did not have enough ability to extract feature points in the space, and thus the whole algorithm failed to locate.

**Figure 14:**
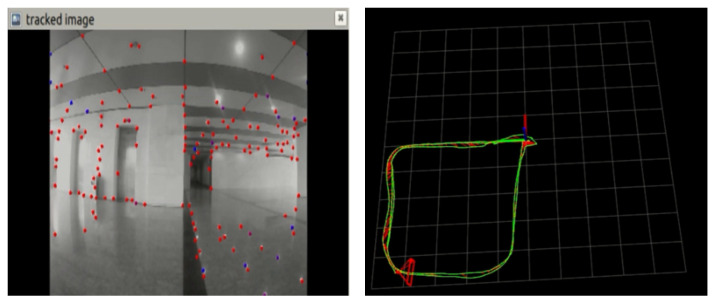
Localization results of algorithm in the paper in a low-texture environment.

As shown in Figure 14, the positioning method used in this paper relied not only on the currently collected image information but also on the image information before history in image processing, which is more robust than the feature point method relying on the current image each time. At the same time, due to the existence of inertial navigation, the algorithm can ensure the normal operation of the whole positioning system by measuring the own movement of the robot, even if there are some moments when the texture is missing.

## 5. Conclusions

In this paper, the traditional visual SLAM algorithm was improved for the characteristics of large personnel flow and sparse background texture in an underground complex. In order to solve the problem that it is difficult for the front end to extract feature points from weak texture and the tracking is easy to be lost, the Harris algorithm with a stronger ability to extract feature points was introduced, which was combined with optical flow pyramid tracking to enhance the robustness of the visual positioning system. Various error sources of IMU were analyzed, and the performance of the Euler method and median method on dynamic model discretization was compared through simulation, and the Euler method with shorter time consumption was selected. Finally, the non-uniform rational B-spline was used to solve the problem of the non-synchronization of sensors with different frequencies, and the tight coupling between the vision sensor and IMU was realized. In the real physical environment, the research group simulated the environment of an urban underground complex, and the experiment showed that the fusion algorithm presented in this paper had better experimental results in terms of positioning accuracy, anti-dynamic interference, and robustness of weak texture than the positioning method using inertial navigation or vision only.

## Figures and Tables

**Table 1:** The parameters in matrix $F_{t}$, $G_{t}$.

Coefficient	Mathematical Formula
$f_{01}$	$-\frac{1}{4}\left(R_{b_{i}b_{k}}{\left[a^{b_{k}}-b_{k}^{a}\right]}_{\times }\delta t^{2}+R_{b_{i}b_{k+1}}{\left[\left(a^{b_{k}}-b_{k}^{a}\right)\right]}_{\times }\left(I-{\left[\omega \right]}_{\times }\delta t\right)\delta t^{2}\right)$
$f_{03}$	$-\frac{1}{4}\left(q_{b_{i}b_{k}}+q_{b_{i}b_{k+1}}\right)\delta t^{2}$
$f_{04}$	$-\frac{1}{4}\left(R_{b_{i}b_{k+1}}{\left[\left(a^{b_{k}}-b_{k}^{a}\right)\right]}_{\times }\delta t^{2}\right)\left(-\delta t\right)$
$f_{11}$	$I-{\left[\omega \right]}_{\times }$
$f_{21}$	$-\frac{1}{2}\left(R_{b_{i}b_{k}}{\left[a^{b_{k}}-b_{k}^{a}\right]}_{\times }\delta t+R_{b_{i}b_{k+1}}{\left[\left(a^{b_{k}}-b_{k}^{a}\right)\right]}_{\times }\left(I-{\left[\omega \right]}_{\times }\delta t\right)\delta t\right)$
$f_{23}$	$-\frac{1}{2}\left(q_{b_{i}b_{k}}+q_{b_{i}b_{k+1}}\right)\delta t$
$f_{24}$	$-\frac{1}{2}\left(R_{b_{i}b_{k+1}}{\left[\left(a^{b_{k}}-b_{k}^{a}\right)\right]}_{\times }\delta t\right)\left(-\delta t\right)$
$v_{00}$	$\frac{1}{4}q_{b_{i}b_{k}}\delta t^{2}$
$v_{01}$	$-\frac{1}{4}\left(R_{b_{i}b_{k+1}}{\left[\left(a^{b_{k}}-b_{k}^{a}\right)\right]}_{\times }\delta t^{2}\right)\left(\frac{1}{2}\delta t\right)$
$v_{02}$	$\frac{1}{4}q_{b_{i}b_{k+1}}\delta t^{2}$
$v_{03}$	$-\frac{1}{4}\left(R_{b_{i}b_{k+1}}{\left[\left(a^{b_{k}}-b_{k}^{a}\right)\right]}_{\times }\delta t^{2}\right)\left(\frac{1}{2}\delta t\right)$
$v_{21}$	$-\frac{1}{2}\left(R_{b_{i}b_{k+1}}{\left[\left(a^{b_{k}}-b_{k}^{a}\right)\right]}_{\times }\delta t^{2}\right)\left(\frac{1}{2}\delta t\right)$
$v_{23}$	$-\frac{1}{2}\left(R_{b_{i}b_{k+1}}{\left[\left(a^{b_{k}}-b_{k}^{a}\right)\right]}_{\times }\delta t^{2}\right)\left(\frac{1}{2}\delta t\right)$

**Table 2:** The comparison of error before and after sensor fusion.

Number	Pure Inertial Odometer			Fusion Algorithm Odometer		
	Origin	Destination	Error	Origin	Destination	Error
1	X: 0.01, Y: 0.04	X: −0.03, Y: −0.05	0.041	X: 0.03, Y: 0.04	X: 0.01, Y: 0.05	0.022
2	X: 0.02, Y: 0.01	X: −0.10, Y: −0.12	0.177	X: 0.02, Y: 0.04	X: 0.04, Y: 0.06	0.028
3	X: 0.05, Y: 0.03	X: −0.13, Y: −0.04	0.193	X: 0.07, Y: 0.02	X: 0.03, Y: 0.05	0.05
4	X: 0.03, Y: 0.02	X: −0.03, Y: −0.04	0.085	X: 0.06, Y: 0.02	X: 0.05, Y: 0.04	0.022
5	X: 0.04, Y: 0.01	X: −0.09, Y: −0.05	0.143	X: 0.02, Y: 0.04	X: 0.03, Y: 0.07	0.032
6	X: 0.02, Y: 0.05	X: −0.03, Y: −0.08	0.139	X: 0.05, Y: 0.04	X: 0.07, Y: 0.03	0.022
Average error	0.130			0.029		

## Data Availability

Not applicable.
